# SGEF is a potential prognostic and therapeutic target for lung adenocarcinoma

**DOI:** 10.1186/s12957-018-1331-8

**Published:** 2018-02-17

**Authors:** Qian Chen, Xiao Lu, Quan-Xing Liu, Dong Zhou, Yuan Qiu, Ji-Gang Dai, Hong Zheng

**Affiliations:** 1 Team four of the Second group, Graduate school, Third Military Medical University (Army medical university), Chongqing, 400037 China; 2 Department of Thoracic Surgery, Xinqiao Hospital, Third Military Medical University (Army medical university), Chongqing, 400037 China; 3 Department of General Surgery, Xinqiao Hospital, Third Military Medical University (Army medical university), Chongqing, 400037 China

**Keywords:** Lung adenocarcinoma, SGEF, Invasion, Poor prognosis

## Abstract

**Background:**

SH3-containing guanine nucleotide exchange factor (SGEF), a RhoG-specific guanine nucleotide exchange factor (GEF), was consider as a key signal that determines cancer cell invasion. Although SGEF has been considered to highly express in glioma and prostate cancer. However, it is not well illustrated in LAC.

**Methods:**

In this experiment, expression of SGEF was detected in 92 LAC and corresponding normal tissue samples by immunohistochemistry. In addition, we evaluated the invasion and migration of lung adenocarcinoma cells by the gain and loss of SGEF expression. Furthermore, RhoG activity was measured by GST pull-down assay.

**Results:**

SGEF is highly expressed in LAC tissues than in normal lung tissues and was associated with the TNM stage. Lung adenocarcinoma patients with low SGEF subgroup had longer overall survival compared to those with high expression. Furthermore, univariate analysis showed that SGEF expression was an independent prognostic factor for overall survival in lung adenocarcinoma. Silencing of SGEF effectively suppressed the invasion and migration of human lung adenocarcinoma cells in vitro by inhibiting RhoG activity, and over-expression of SGEF could reverse this phenomena.

**Conclusion:**

SGEF is a novel prognostic target in human lung adenocarcinoma.

**Electronic supplementary material:**

The online version of this article (10.1186/s12957-018-1331-8) contains supplementary material, which is available to authorized users.

## Background

LAC is the familiar common histological subtype of non-small-cell lung cancer (NSCLC), which is the first cause of cancer-related deaths worldwide [1]. Although lung cancer mortality rate has been decreasing owing to advances made in the treatment of lung adenocarcinoma (LAC), the prognosis of advanced lung adenocarcinoma is very poor because of its invasion and metastasis [2]. Therefore, it is worthwhile for us to have a better understanding of the molecular mechanism of invasion and migration of LAC and find a more sensitive and novel target for predicting the prognosis.

Recently, about 22 Rho family members were identified to be crucial for forming filopodium [3], which are demonstrated embedded in or protruding from the lamellipodial actin network to promote lung cancer cell migration and invasion [4]. The activity of Rho GTPases was regulated by Rho guanine nucleotide exchange factors that switch bound GDP to bound GTP [5], indicating that some GEF factors may act as oncogene by activating Rho activation.

SGEF is a RhoG-specific guanine nucleotide exchange factor (GEF) [6]. As shown in Additional file 1: Figure S1, its full length contains an amino-terminal proline-rich region (Pro), a Dbl homology (DH) domain, and pleckstrin homology (PH) domain, as well as Src homology 3 (SH3) domain [7]. DH-PH domain is thought to be correlated with protein-protein interactions and a center of exchange SGEF activation [7]. For instance, the domains of DH/PH in SGEF strongly exchange RhoG [8] and interact with HPV E6 oncoprotein which contains PDZ domain, accelerating invasive phenotype of the HPV-induced cervical cancer. Furthermore, EGFR stability was meditated by SGEF through inhibited EGFR trafficking promoting prostate cancer cell progression. Overall, SGEF is complex and multivariate in the cancer progression. However, there is no information about the functional and clinical value of SGEF in LAC. Therefore, it deserves to explore the SGEF expression and function in LAC.

In summary, we showed that SGEF is highly expressed in cancer than in adjacent cancer and its expression was associated with invasion depth and lymph node metastasis. Furthermore, we found that silencing SGEF in lung cancer cell leads to less invasive phenotype by regulating RhoG activity. Clinically, our data indicated that SGEF may be a novel prognostic marker in LAC.

## Methods

### Cell culture, siRNA, and plasmid transfection

H1975 and H1299 cell lines were purchased from the American Type Culture Collection, which were maintained in Dulbecco’s modified Eagle’s medium (DMEM; Gibco) containing 10% fetal bovine serum (BI). For SGEF siRNAs, H1975 cell lines were infected with siRNA derived from Gene Pharma and were resynthesized: SiCtrl sequences: (5′-UUCUCCGAACGUGUCACGUTT-3′); SiSGEF sequences: (5′-GGAAAUUUCCTTUCCUCTAAT-3′). For SGEF over-expression analysis of H1299 cells, the coding sequence of SGEF was amplified from H1299 cDNA and cloned into pcDNA3.1 (+) plasmid by using In-Fusion HD Cloning Kit (TAKARA). The primers used for PCR were as follows: Fw: 5′-TACCGAGCTCGGATCCATGGACGGCGAGAGCGAGG-3′; Rv: 5′-CTGGATATCTGCAGAACTACACGTTGGTCTCCAG-3′. Lipofectamine® 2000 (Invitrogen) was used for plasmid transfection.

### Clinical samples

The tissue microarray including 92 tumor tissues and 87 corresponding non-cancerous tissue specimens was obtained from lung adenocarcinoma patients diagnosed and operated at the Xinqiao Hospital, Third Military Medical University, from 2008 to 2013 and followed up until 2016. A total of 13 patients were lost to follow up among these patients. None of the patients had received radiotherapy or chemotherapy before surgery.

### Immunohistochemistry and scoring

Immunohistochemistry (IHC) detection of SGEF was performed using the Dako Envision FLEX+ system. Tissue samples embedded in paraffin were sectioned, deparaffinized, and subjected to antigen retrieval performed in citrate buffer (pH 6.0). Slides were incubated at 4 °C overnight with the SGEF antibody (1:500; Abcam) and then with a secondary HRP-conjugated antibody (Dako) at 37 °C. Sections were stained with 3-diaminobenzidine (DAB) for 2 min and scored for the extent of SGEF expression using the following system: 0, 0–5% SGEF-1-positive cells; 1, < 25% positive cells; 2, 25–50% positive cells; 3, 50–75% positive cells; and 4, 75–100% positive cells. The staining intensity was scored as follows: 0, no positive staining; 1, weak staining; 2, moderate staining; and 3, strong staining. The final scores were obtained by multiplying the extent scores by intensity scores (0, 1, 2, 3, 4, 6, 8, 9, or 12) and analyzed using the statistical X-tile software with 4 as the cutoff value.

### Detection of EGFR status

Increased EGFR gene copy number was assessed by standard FISH analysis. Patients were considered to be FISH positive if they displayed high polysomy (greater than or equal to four copies of the EGFR gene in greater than or equal to 40% of cells) or gene amplification, defined by the presence of tight EGFR gene clusters and a gene/chromosome per cell ratio of at least 2, or an average of greater than or equal to 15 copies of EGFR per cell in greater than or equal to10% of analyzed cells.

### Biostatistics mining of NCBI database

The LAC patients’ mRNA expression microarray data were downloaded from GDS2771 in NCBI GEO. There are 90 normal smokers not diagnosed with lung cancer and 97 smokers with lung cancer. For the survival analysis of SGEF, we use online (http://kmplot.com/analysis/index.php?p=service&cancer=lung) to generate Kaplan–Meier curves, which include 866 gastric cancer patients with available clinical data. The median expression was used as the final cutoff value.

### Western blotting

Western blotting was investigated as previously described [9]. The primary antibodies were used as follows: SGEF (1:400; Abcam), antibody against GAPDH (1:1000 CST), and antibodies against RhoG (1:1000 CST).

### Reverse transcription and quantitative real-time PCR

Total RNA was isolated as previously described [9]. cDNA was synthesized from 500 ng of total RNA in a 20-μL reaction volume using the SuperScript III First-Strand Synthesis SuperMix Kit (Invitrogen) for 30 min at 37 °C, followed by 90 °C for 1 min, and the primers for RT-qPCR analysis were SGEF (sense: 5′-TGC TGA AAG GAC AAG GAA CA-3′; anti-sense: 5′-GTAGTTTTGATACAGGACAGCATT-3′) and GAPDH (sense, 5′-TGTTCGTCATGGG--GTGAAC-3′, anti-sense: 5′-ATGGCATGGACTGTGGTCAT-3′).

### Rho GTPase activation assays

The activation of RhoG proteins was performed as described previously [7]. Briefly, pull-down assays were done using purified GST-ELMO with cell extracts from LAC cells transfected with SiSGEF and SiCtrl. Cellular extracts were prepared from by lysing the cells in 250 ml of 50 mM Tris, pH 7.4, 10 mM MgCl2, 500 mM NaCl, 1% Triton X-100, 0.1% SDS, 0.5% deoxycholate, and protease inhibitors and then equalized for protein concentration. Supernatants were incubated with purified GST-ELMO conjugated to glutathione sepharose beads after clearing it at 12,000 g. RhoG-GTP was investigated by WB and compared with the total RhoG present within the same cell lysate.

### Invasion and migration assays

For the migration assay, H1975 cell was plated at the density of 2 × 10^4^ cells/well in serum-free DMEM in the upper chamber of 24-well transwell plates containing 8.0-μm-pore Millicell inserts, while the lower chambers were filled with DMEM supplemented with 10% FBS as a chemotaxis agent. For the invasion assay, Millicell inserts were coated with 1 mg/mL of Matrigel (BD Biosciences). Cells invaded/migrated to the lower membrane surface were counted under a light microscope at × 200 magnification in at least four randomly selected fields, and the average number of cells per field was calculated. All the experiments were performed in triplicate.

## Results

### SGEF is over-expressed in lung cancer

**Fig. 1:**
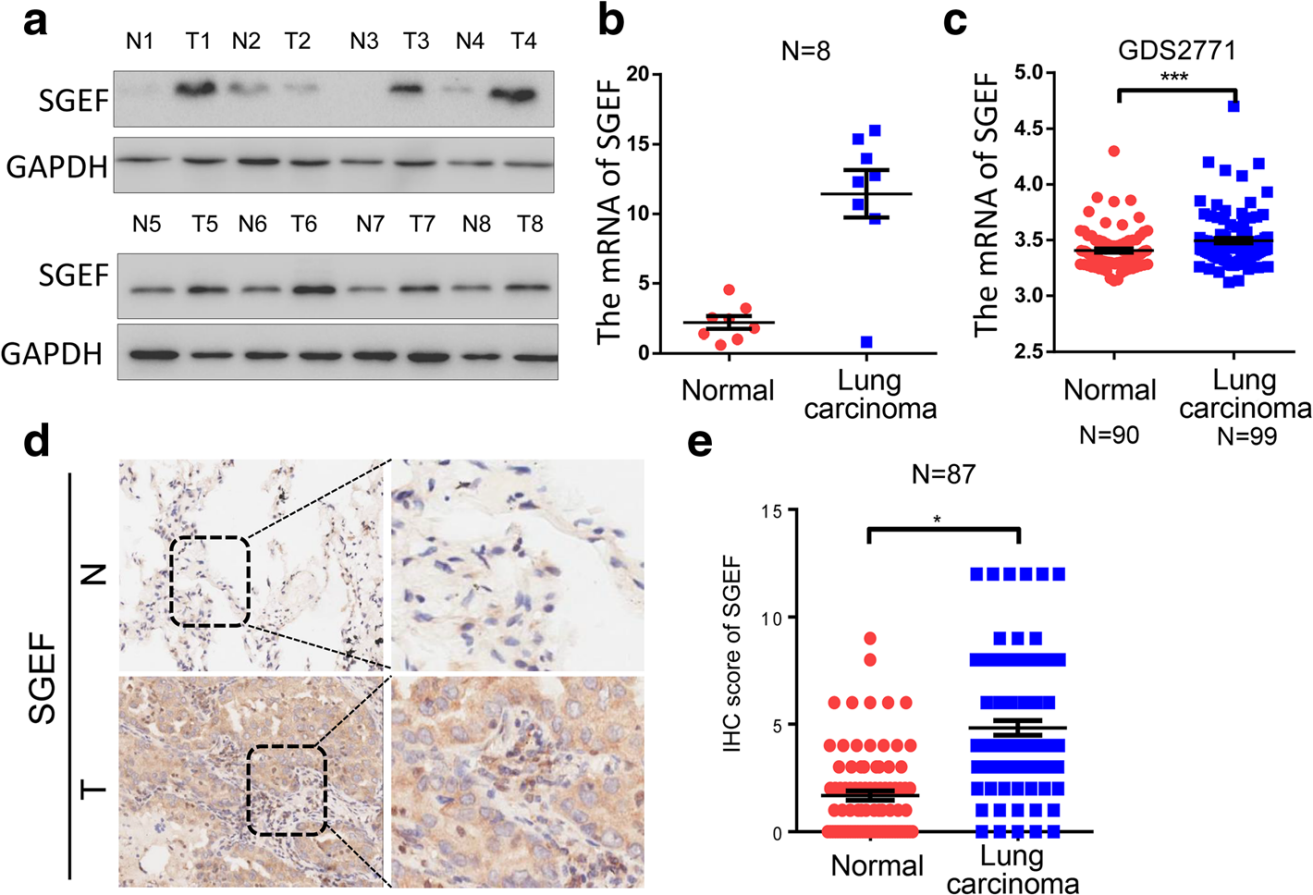
SGEF is highly expressed in human LAC tissues than in corresponding non-tumor tissues. **a**, **b** The protein (**a**) and mRNA (**b**) expression of adjacent normal tissues and matching cancerous tissues in eight LAC specimens. **c** The mRNA of SGEF in 90 normal lung tissues and 99 lungs carcinoma tissues from GEO database. **d** Representative images of SCIN staining (brown color = 50 μm) in normal lung and LAC samples. **e** IHC scores of normal tissues and cancerous tissues in 87 paired LAC specimens

In order to explore the expression of SGEF in LAC, we first investigate eight pairs of lung carcinoma specimens and corresponding neighboring non-cancerous tissues by western blotting and PCR. We found that SGEF is highly expressed in lung carcinoma than in adjacent tissues, regardless of protein (Fig. 1a) or mRNA (Fig. 1b). Consistent with our previous result, we found that the same result was testified in GEO database (Fig. 1c).Furthermore, SGEF expression were investigated in 87 pairs of lung carcinoma specimens and corresponding neighboring non-cancerous tissues. We found that SGEF was mostly localized in the cell cytoplasm, and the proportion of cells with high SGEF expression was significantly higher in the lung adenocarcinoma tissues (58.7%, 54/92) than in the adjacent normal tissues (17.24%, 15/87) (Fig. 1d and Table 1). Overall, we found that SGEF is highly expressed in lung carcinoma than in matched adjacent tissues.

**Table 1:** SGEF expression in LAC and adjacent normal mucosa

	SGEF (low)	SGEF (high)	*P* value
Carcinoma	38	54	*P* < 0.001
Adjacent normal tissue	72	15	

### High SGEF is closely related with advanced TNM stage and poor prognosis in LAC

**Fig. 2:**
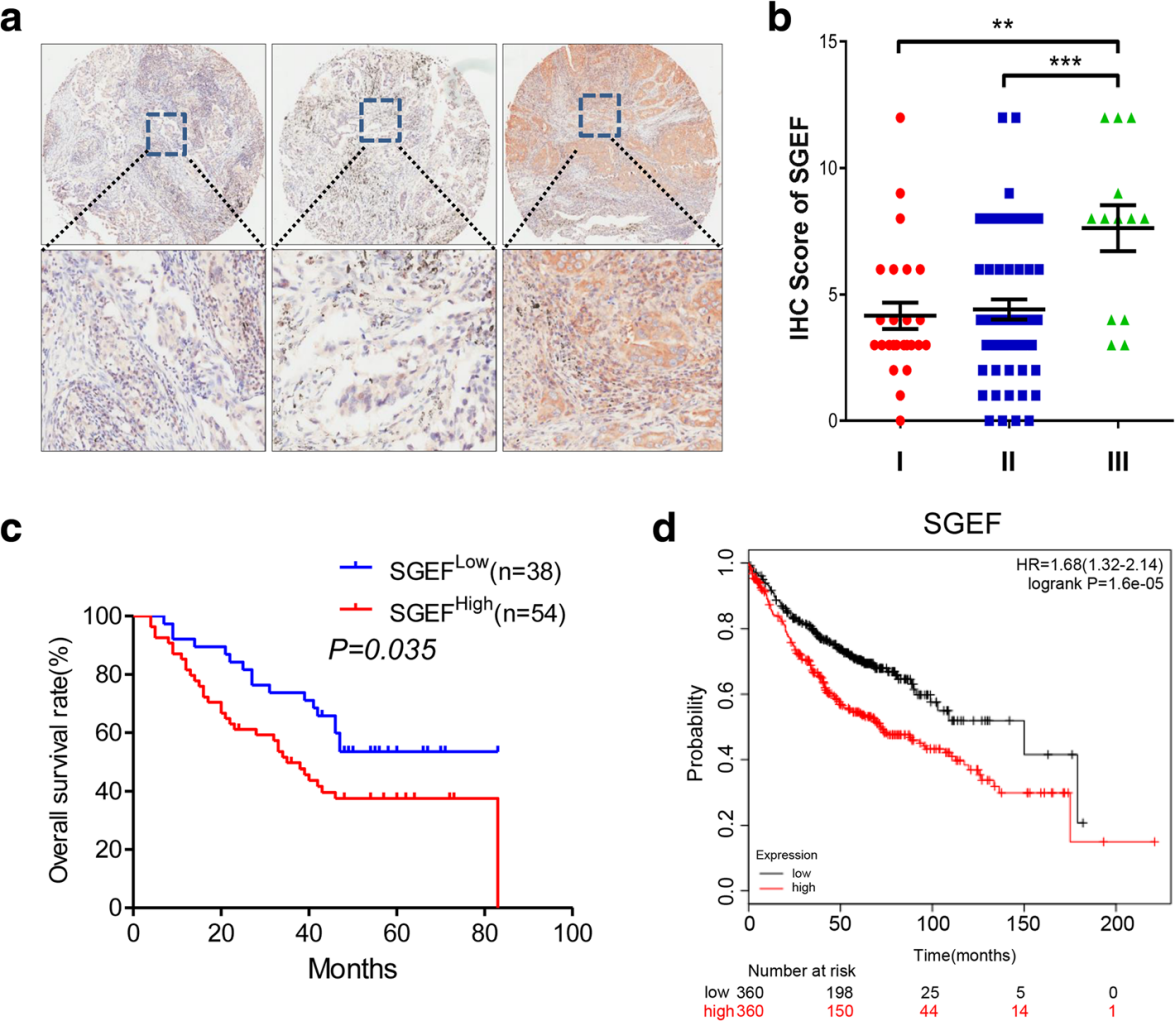
SGEF expression is correlated with TNM stage and poor prognosis. **a** Representative images of SGEF staining in LAC specimen at different TNM stages. Magnification in the upper panel: × 40 (scale bar = 200 μm); magnification in the right panel: × 200 (scale bar = 20 μm). **b** IHC scores of SGEF expression in tumor of all stages. The expression of SGEF in TNM III stage was significantly higher than that in early TNMΙ and TNMΠ stages. **c** Kaplan–Meier analyses of 92 CRC patients showing that high-SGEF patients (*n* = 54) have shorter overall survival as compared with low-SGEF patients (*n* = 38). **d** Patients with highly expressed SGEF has a relatively higher risk of mortality (*P* < 0.001) from GEO databases

We next investigated the potential relationship of SGEF expression and clinical pathological parameters. The pathological features of LAC were summarized in Table 2. SGEF expression is positively related with disease stage (Fig. 2a, b, Table 3), tumor depth (*P* = 0.026), and lymphatic metastasis (*P* = 0.021). Furthermore, overall survivals for patients with low SGEF were significantly lower than those with high-SGEF tumors (Fig. 2c, *P* < 0.001). Additionally, similar results were observed in GEO database (Fig. 2d, *P* < 0.001). Univariate analysis showed that SGEF was an independent prognostic factor in LAC (HR = 1.846; 95% CI 1.029–3.310; *P* = 0.040, Table 4). Therefore, SGEF seems to serve as a predictor for the prognosis of patients with LAC.

**Table 2:** The clinical features of the LAC specimens used in this study

	WHO grade		
Feature	I (*n* = 11)	II (*n* = 73)	III (*n* = 8)
Gender			
Male	7	38	6
Female	4	35	2
Age at diagnosis			
<60	5	25	3
≥ 60	6	48	5
*T* stage			
*T*_1–2_	11	54	4
*T*_3–4_	0	19	4
*N* stage			
*N*_0_	9	34	4
*N*_1–3_	2	39	4
TNM stage			
I	8	25	2
II	3	22	2
III	0	26	4
Location			
Left	3	26	4
Right	8	47	4

**Table 3:** The relationship between SGEF expression and clinicopathological features of LAC patients

	SGEF		
Feature	High (*n* = 54)	Low (*n* = 38)	*P* value
Gender			*P* = 0.192
Male	33 (61.11%)	18 (47.37%)	
Female	21 (28.89%)	20 (52.63%)	
Age at diagnosis			*P* = 0.781
<60	20 (37.04%)	13 (34.21%)	
≥ 60	34 (62.96%)	25 (65.79%)	
Location			*P* = 0.407
Left	21 (38.89%)	12 (31.58%)	
Right	33 (61.11%)	26 (68.42%)	
EGFR status			*P* = 0.545
Mutant	18 (33.33%)	15 (39.47%)	
Wild type	36 (66.67%)	23 (60.53%)	
*T* stage			*P* = 0.028
*T*_1–2_	36 (66.66%)	33 (86.84%)	
*T*_3–4_	18 (33.34%)	5 (13.16%)	
*N* stage			*P* = 0.011
*N*_0_	21 (38.88%)	25 (65.79%)	
*N*_1–3_	33 (61.12%)	13 (34.21%)	
TNM stage			*P* < 0.001
I	7 (12.96%)	18 (47.36%)	
II + III	47 (87.04%)	20 (52.63%)	
Histological grade			*P* = 0.608
Well	6 (11.11%)	5 (11.36%)	
Moderate	42 (77.78%)	31 (70.45%)	
Poor	6 (11.11%)	2 (4.54%)

**Table 4:** Univariate and multivariate analysis for overall survival in LAC

Factors	Univariate		Multivariate	
	HR (95% CI)	*P* value	HR (95% CI)	*P* value
Gender	0.666 (0.379–1.169)	0.156	0.578 (0.321–1.042)	0.069
Age	1.019 (0.996–1.042)	0.839	1.045 (1.010–1.081)	0.011
Location	0.993 (0.527–1.651)	0.811	1.127 (0.587–2.164)	0.718
SGEF expression	1.846 (1.029–3.310)	0.040	1.126 (0.631–2.382)	0.547
Grade	2.474 (1.351–4.532)	0.003	2.337 (1.177–4.643)	0.015
TNM stage	1.603 (1.159–2.217)	0.004	1.532 (1.052–2.202)	0.026

### SGEF accelerates the mobility of LAC cell by activating RhoG

**Fig. 3:**
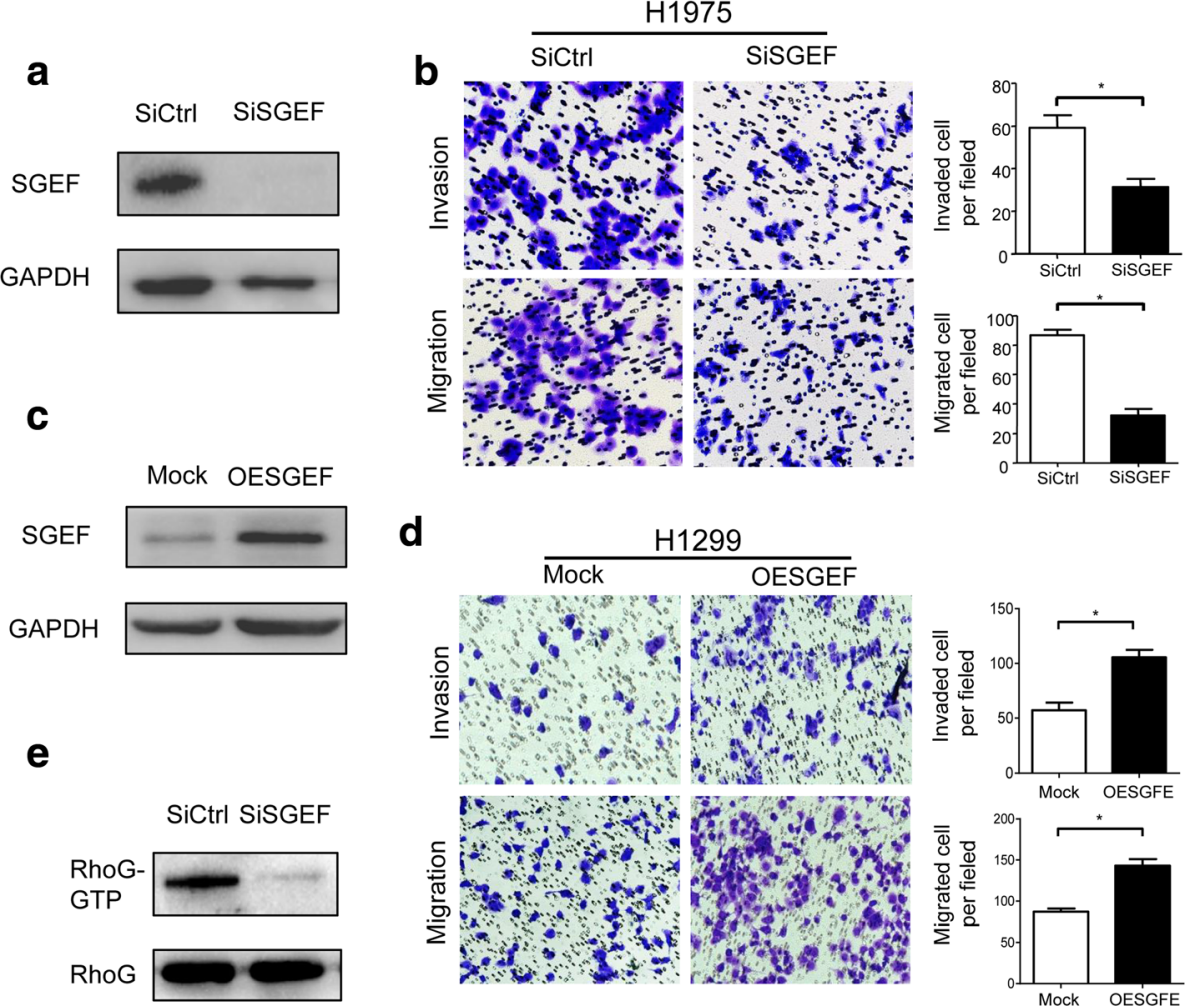
SGEF promotes the invasion of LAC cells in vitro by activating RhoG. **a** Western blotting of SGEF proteins in SiCtrl and SiSGEF in H1975 cell line. **b** Transwell assays established using SiCtrl and SiSGEF H1975 cells, image, and histograms respectively showed the number of cells invading and migrating through the inserts. **c** Western blotting of SGEF proteins in Mock and OESGEF in H1299 cell line. **d** Transwell assays established using Mock and OESGEF H1975 cells, image, and histograms respectively showed the number of cells invading and migrating through the inserts. **e** The RhoG activation in SiSGEF and SiCtrl LAC cell

As SGEF is correlated with invasion depth and lymphatic metastasis, we want to examine the oncogenic function of SGEF in LAC invasion by using LAC cell H1975 with siRNA to generate SGEF knockdown cells (Fig. 3a). A transwell assay showed that treatment with SiSGEF in H1975 cells significantly reduced its motile ability (Fig. 3b), while over-expressed SGEF in H1299 cells increased their mobility (Fig. 3c, d). Furthermore, the activating RhoG was also decreased in H1975-siSGEF cell (Fig. 3e). Taken together, SGEF promotes invasion and migration by inhibiting RhoG activity.

## Discussion

LAC is currently the major histological subtype of lung cancer, and the average of 5-year survival rate was only 15% [10], owing to the invasion and metastasis. Thus, searching for specific invasive therapeutic target was crucial for treating LAC. SGEF was highly expressed in several malignant cancers, such as glioma, HPV-induced cervical cancer, and prostate tumors [11–13]. Herein, we showed that high SGEF expression in LAC is correlated with TNM stage and poor prognosis. Furthermore, we found that SGEF regulated the invasion of LAC cell line by activating RhoG via siRNA. Our result revealed that SGEF may be a novel prognostic and therapeutic target in LAC.

Rho GTPases were first identified to promote cell migration and invasion about 20 years ago [14]. RhoG, a subfamily of Rho GTPases, is induced to form actin-rich lamellipodia protrusions and derived cell movement by activating Rac1 pathway. Owing to the RhoG role in regulating various cellular processes [15], downregulation of the RhoG pathway is assumed to be crucial for molecular targeting therapy. These facts suggest that some guanine nucleotide exchange factors (GEF), upregulator proteins of Rho GTPases, may be involved in the cancer progression. Furthermore, several GEFs including PREX1 and Vav2 as well as Vav3 are reported to be over-expressed and associated with carcinoma progression [16, 17]. Consistent with previous study, we showed that SGEF is upregulated in LAC tissue as compared with that in adjacent normal tissue and was positively associated with TNM stage. Most importantly, SGEF is an independent prognostic factor in LAC and predicts poor prognosis. These findings support the notion that some GEF protein was involved in the progression of cancer.

Recent studies have shown that SGEF was involved in the response of glioma cells to TMZ treatment modulates the DNA repair. Furthermore, SGEF promotes prostate cancer cell progression by interacting with Grb2 for activating ERK pathway and enhancing EGFR stability [18, 19]. Herein, we showed that SGEF promotes the invasion and migration of LAC by regulating the activity of RhoG via silencing SGEF expression. These facts indicate that SGEF was involved in the progression of tumor via various pathways, highlighting the importance and complexity of SGEF.

In summary, we investigated the function of SGEF in LAC initiation and progression. Our study indicates that SGEF was a novel prognostic target in LAC. Our data provide a new insight into the mechanism responsible for the development of human LAC.

## Conclusions

We showed that SGEF expression in LAC tissues was higher than that in normal tissues and was associated with the TNM stage. Furthermore, our data indicate that SGEF may be a potential marker for the prediction of prognosis in LAC. Functionally, we found that SGEF promotes invasion and migration by switching RhoG-GDP to RhoG-GTP.

## Additional file

Additional file 1: **Figure S1.** The schematic of the SGEF protein structure. Full length of SGEF protein contains an amino-terminal proline-rich region (Pro), a Dbl homology (DH) domain, and pleckstrin homology (PH) domain, as well as Src homology 3 domain (SH3). (TIFF 58 kb)

## References

[1] Torre LA, Bray F, Siegel RL, Ferlay J, Lortet-Tieulent J, Jemal A (2015). Global cancer statistics. CA Cancer J Clin.

[2] Takamochi K, Oh S, Matsunaga T, Suzuki K (2017). Prognostic impacts of EGFR mutation status and subtype in patients with surgically resected lung adenocarcinoma. J Thorac Cardiovasc Surg.

[3] Burridge K, Wennerberg K (2004). Rho and Rac take center stage. Cell.

[4] Mattila PK, Lappalainen P (2008). Nat Rev Mol Cell Biol.

[5] Schmidt A, Hall A (2002). Guanine nucleotide exchange factors for Rho GTPases: turning on the switch. Genes Dev.

[6] Patel JC, Galan JE (2006). Differential activation and function of Rho GTPases during Salmonella-host cell interactions. J Cell Biol.

[7] Ellerbroek SM, Wennerberg K, Arthur WT, Dunty JM, Bowman DR, Demali KA, Der C, Burridge K (2004). SGEF, a RhoG guanine nucleotide exchange factor that stimulates macropinocytosis. Mol Biol Cell.

[8] Rehak S (1987). Czech and Slovak professors of ophthalmology. Cesk Oftalmol.

[9] Liu QX, Zheng H, Deng XF, Zhou D, Dai JG (2015). Status of the Parkinson's disease gene family expression in non-small-cell lung cancer. World J Surg Oncol.

[10] Xue M, Tao W (2017). Upregulation of MUC1 by its novel activator 14-3-3zeta promotes tumor invasion and indicates poor prognosis in lung adenocarcinoma. Oncol Rep.

[11] Wang H, Wu R, Yu L, Wu F, Li S, Zhao Y, Li H, Luo G, Wang J, Zhou J (2012). SGEF is overexpressed in prostate cancer and contributes to prostate cancer progression. Oncol Rep.

[12] Visentini M, Conti V, Cagliuso M, Siciliano G, Scagnolari C, Casato M, Fiorilli M (2012). Persistence of a large population of exhausted monoclonal B cells in mixed cryoglobuliemia after the eradication of hepatitis C virus infection. J Clin Immunol.

[13] Goicoechea S, Zinn A M, Awadia SS, Snyder K, Garcia-Mata R (2017). A RhoG-mediated signaling pathway that modulates invadopodia dynamics in breast cancer cells. J Cell Sci.

[14] Ridley AJ, Comoglio PM, Hall A (1995). Regulation of scatter factor/hepatocyte growth factor responses by Ras, Rac, and Rho in MDCK cells. Mol Cell Biol.

[15] Polat R, Peker K, Guloksuz CT, Ergil J, Akkaya T (2015). Comparison of the postoperative analgesic effects of paracetamol-codeine phosphate and naproxen sodium-codeine phosphate for lumbar disk surgery. Kaohsiung J Med Sci.

[16] Schendel SA, Peauroi J (2009). Magnesium-based bone cement and bone void filler: preliminary experimental studies. J Craniofac Surg.

[17] Barrio-Real L, Kazanietz MG (2012). Rho GEFs and cancer: linking gene expression and metastatic dissemination. Sci Signal.

[18] Wang H, Li S, Li H, Li C, Guan K, Luo G, Yu L, Wu R, Zhang X, Wang J, Zhou J (2013). SGEF enhances EGFR stability through delayed EGFR trafficking from early to late endosomes. Carcinogenesis.

[19] Wang H, Li S, Li H, Wang P, Huang F, Zhao Y, Yu L, Luo G, Zhang X, Wang J, Zhou J (2014). Grb2 interacts with SGEF and antagonizes the ability of SGEF to enhance EGF-induced ERK1/2 activation. Mol Cell Biochem.

