# Supplementary figures and images for: SGEF is a potential prognostic and therapeutic target for lung adenocarcinoma
Source: World J Surg Oncol. 2018 Feb 17;16:32. doi: 10.1186/s12957-018-1331-8 (PMC5816374; doi:10.1186/s12957-018-1331-8)

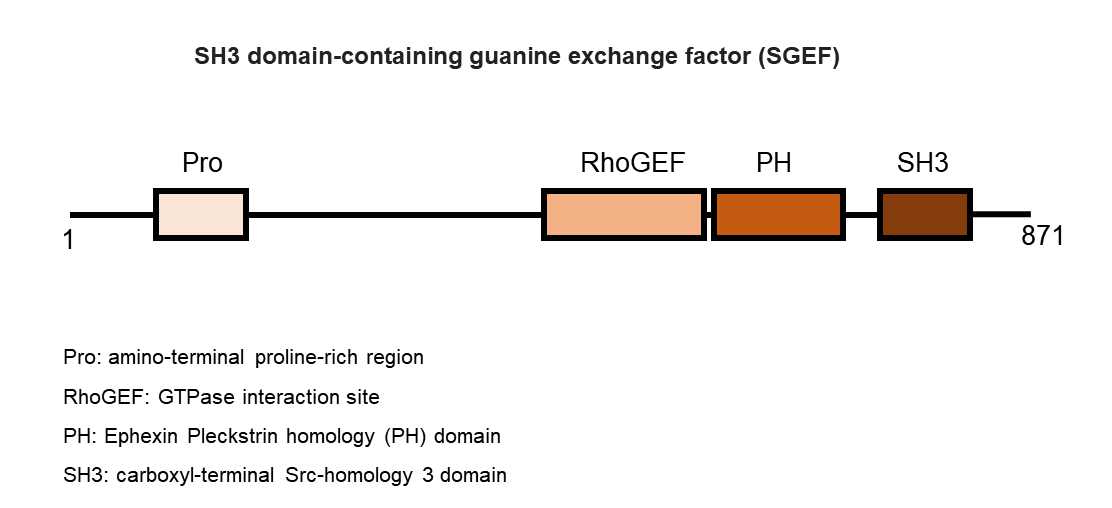

Supplement: Supplementary file 1 — Figure S1. The schematic of the SGEF protein structure. Full length of SGEF protein contains an amino-terminal proline-rich region (Pro), a Dbl homology (DH) domain, and pleckstrin homology (PH) domain, as well as Src homology 3 domain (SH3). (TIFF 58 kb) [file 12957_2018_1331_MOESM1_ESM.tif]
